# Asymptomatic Bone Cement Pulmonary Embolism after Vertebroplasty: Case Report and Literature Review

**DOI:** 10.1155/2013/591432

**Published:** 2013-05-07

**Authors:** Girolamo Geraci, Giorgio Lo Iacono, Chiara Lo Nigro, Fabio Cannizzaro, Massimo Cajozzo, Giuseppe Modica

**Affiliations:** Section of General and Thoracic Surgery, University of Palermo, Via Liborio, Giuffrè 5, 90124 Palermo, Italy

## Abstract

*Introduction*. Acrylic cement pulmonary embolism is a potentially serious complication following vertebroplasty. *Case Report*. A 70-year-old male patient was treated with percutaneous vertebroplasty for osteoporotic nontraumatic vertebral collapse of L5-S1. Asymptomatic pulmonary cement embolism was detected on routine postoperative chest radiogram and the patient was treated with enoxaparin, amoxicillin, and dexamethasone. At the followup CT scan no further migration of any cement material was reported; and the course was uneventful. *Discussion*. The frequency of local leakage of bone cement is relatively high (about 80–90%), moreover, the rate of cement leakage into the perivertebral veins (seen in up to 24% of vertebral bodies treated) with consequent pulmonary cement embolism varies from 4.6 to 6.8% (up to 26% in radiologic studies); the risk of embolism is increased with the liquid consistency of the cement and with the treatment of some malignant lesions. Patients may remain asymptomatic and develop no known long-term sequelae. *Conclusions*. Our ancedotal case illustrates the need for close monitoring of patients undergoing percutaneous vertebroplasty and emphasizes the importance of prompt and correct diagnosis and treatment, even if actually there is no agreement regarding the therapeutic strategy.

## 1. Introduction

Bone cement embolism is a severe and potentially life-threatening complication of cement (polymethylmethacrylate, PMM) vertebroplasty.

We report a case of asymptomatic PMM pulmonary embolism following a surgical vertebroplasty.

## 2. Case Report

A 70-year-old male patient with a complex medical history of coronary heart disease and hypertension (bicameral pace-maker dependent, left carotid artery stent, and triple aortocoronary bypass) was admitted to our university hospital for osteoporotic nontraumatic vertebral collapse of L5-S1 and spondylotic degeneration of vertebral bodies.

Preoperative serum chemistries and electrocardiogram were normal.

The patient was in a prone position and percutaneous vertebroplasty was performed with a 10-gauge needle, under biplane fluoroscopic control with unilateral transpedicular approach.

Bone cement was classically prepared at 24°C by mixing 30 mL of the PMM powder with 2 g of sterile barium sulfate powder for opacification and 1 g of powdered antibiotic, before adding 10 mL of the liquid monomer; cement injection was monitored on continuous fluoroscopy in the lateral plane with intermittent evaluation in anteroposterior projection to detect early lateral venous leaks. When cement was visualized in the posterior fourth of the vertebral body or beyond the confines of the vertebral body, the procedure was terminated.

Postoperative serum chemistries, arterial blood gas, and cardiac enzymes were normal, and the postoperative course was uneventful.

**Figure 1:**
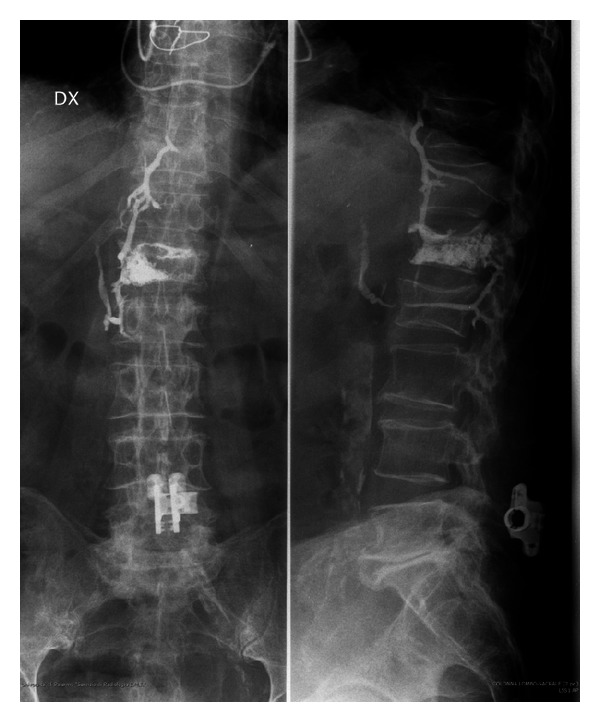
Cement leakage into the Batson's paravertebral venous system.

**Figure 2:**
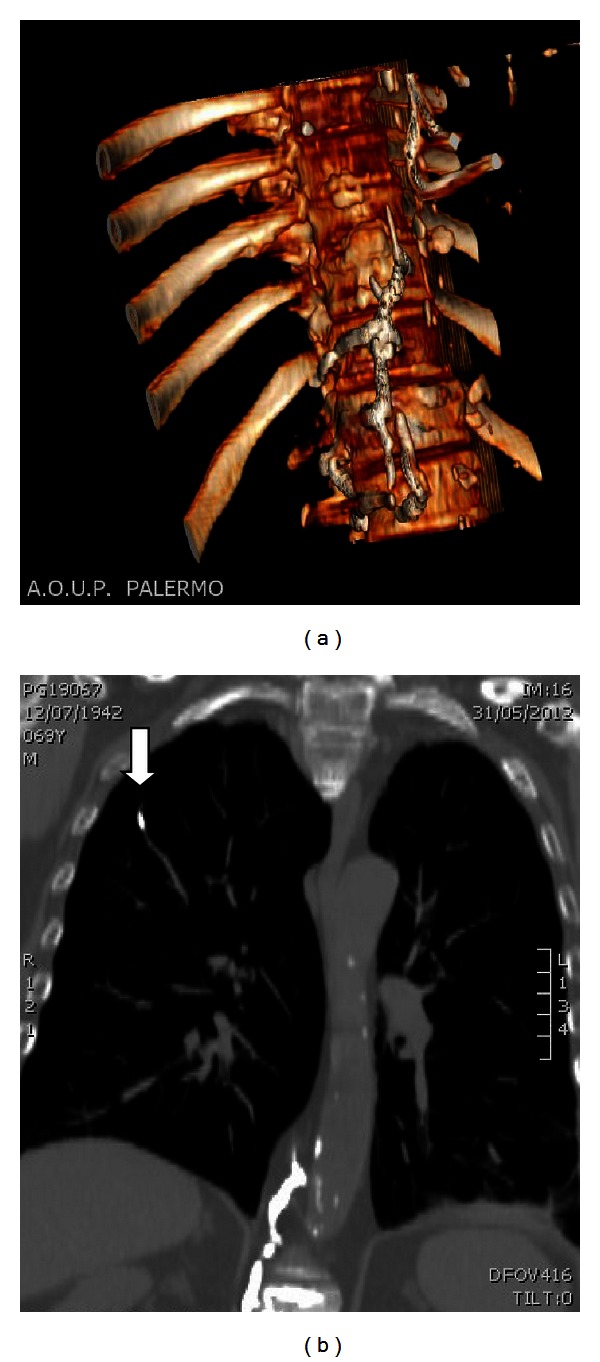
(a) Cement leakage into the Batson's paravertebral venous system. (b) Pulmonary embolism (arrow: presence of cement in the subsegmentary and segmentary pulmonary arteries of the right superior lobe).

Pulmonary cement embolism was detected on routine postoperative chest radiograph (Figure 1, cement leakage into the Batson's paravertebral venous system) and confirmed with chest computed tomography (Figures 2(a) and 2(b)).

Thoracic CT revealed the characteristic appearance of cement leakage at the level of the vertebroplasty in the perivertebral venous system, draining into juxtarenal inferior cava vein, partially obstructed by the same material, and into the azygos system between D9 and L2; the presence of cement in the subsegmentary and segmentary pulmonary arteries of the right superior lobe confirmed a pulmonary microembolism (centimetric areas of shaded ground glass) caused by cement.

The patient was treated with anticoagulants (enoxaparin 4000 IU a day), antibiotics (Amoxicillin 2 g a day), and corticosteroids (dexamethasone 4 mg a day) and responded favorably.

Prior to discharge on postoperative day 4, a repeat CT scan showed no substantial change in the distribution of the cement. Therefore, he was discharged home on oral warfarin for chronic anticoagulation (to reduce the risk of thrombosis on the cement remaining in the distal part of the arterial pulmonary tree), and monthly follow up was scheduled.

**Figure 3:**
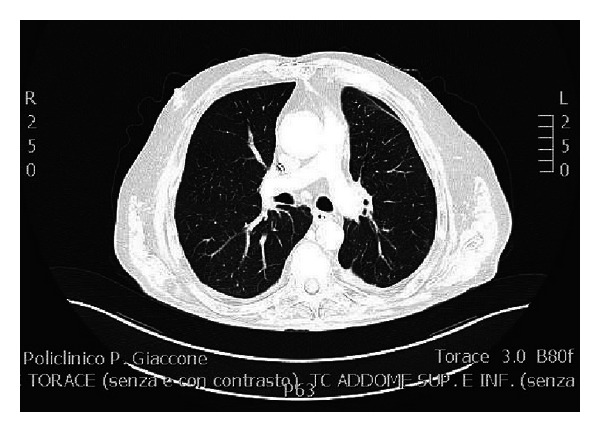
Normal followup CT scan.

The followup CT scan showed no further migration of any cement material. Furthermore, no foreign material was identified within the right atrium-internal vena cava junction (Figure 3).

## 3. Discussion

Bone cement has been widely used in orthopaedic procedure and neurosurgery since 1987: PMM cement, a rapidly setting bone cement, is injected using a transpedicular or paravertebral approach, under fluoroscopic guidance [1].

Although percutaneous vertebroplasty is a relatively safe, simple, and commonly performed procedure for the management of vertebral compression fractures, it can be associated with fatal complications, such as spinal cord compression resulting in paraplegia, cerebral embolism, penetration of the right ventricle, renal artery embolism, and acute respiratory distress syndrome [2]; minor complications, reported in large series, were rare, local, and temporary and included infection, radicular pain, and spinal cord compression; moreover, most complications involved transitory worsening of pain or chest discomfort, dyspnea, and fever [3]; these symptoms may also lead to cardiovascular collapse and, rarely, to death.

The frequency of local leakage of bone cement is relatively high (about 80–90%); moreover, the rate of cement leakage into the perivertebral veins (seen in up to 24% of vertebral bodies treated) with consequent pulmonary cement embolism varies from 4.6 to 6.8% (up to 26% in radiologic studies): the risk is increased with liquid consistency of the PMM and with the treatment of some malignant lesions because of the more frequent cortical destruction of the vertebral body and higher vascularization associated with some malignant tumors; pulmonary embolism is attributable to the passage of the PMM into the perivertebral veins and from there into the azygos vein and the inferior vena cava, to end up in the pulmonary vasculature [3–7].

Otherwise, more reviews in the literature published specifically report with no case of cement pulmonary embolism despite the significant numbers of cement leaks into the venous system (Table 1).

Patients may remain asymptomatic and develop no known long-term sequelae (when cement emboli are encountered in an asymptomatic patient, they are probably of no clinical significance and have no known long-term sequelae). However, when emboli are discovered incidentally on a conventional chest radiograph, their suggestive appearance is a high-density opacity in a tubular branching pattern, corresponding to pulmonary arterial distribution [3, 8].

Some authors reported the use of a preinjection venogram to decrease the incidence of pulmonary embolism, and the injection of sclerosing agents into the vertebral body before vertebroplasty has also been suggested to close venous channels [2, 7].

The treatment for symptomatic or central pulmonary cement embolism is surgical embolectomy, or, in selected cases, percutaneous removal, whereas more conservative management with anticoagulants, antibiotics, and corticosteroid is reserved for smaller or peripherally located emboli [3, 9]: anticoagulation therapy reduces the risk of thrombus formation on the embolic material but cannot reduce the right ventricle afterload and cannot improve the pulmonary ventilation-perfusion ratio, which is the cause of respiratory failure.

## 4. Conclusions

This case allows us to conclude that the risk of pulmonary embolism of PMM might be underestimated. We confirm the necessity of routine chest radiograph following every vertebroplasty in order to detect pulmonary PMM embolism and thereby prevent serious delayed cardiopulmonary failures.

From our brief literature review, it is clear that no agreement has been reached regarding the therapeutic strategy to be used for pulmonary embolism caused by cement, especially in asymptomatic patients, varying from clinical followup to combined heparin plus warfarin.

Surely, pulmonary embolectomy is a therapeutic tool in critical patients and appears to be a reliable and effective procedure in cases of severe respiratory and cardiac failure due to acrylic cement embolism in the main pulmonary trunks because it is the only treatment that could provide complete recovery from pulmonary and cardiac failure.

Anticoagulants appear to have been effective in preventing pulmonary infarction and improving clinical course although it is imprudent to recommend any therapeutic approach based on a single case.

## Figures and Tables

**Table 1:** Case reports and series of patients with asymptomatic pulmonary embolism after vertebroplasty, from PubMed database, queries: “complication of vertebroplasty,” “bone cement pulmonary embolism” (modified and ampliated from [7]).

Authors	No. of asymptomatic pulmonary embolism	Procedure and indications	Therapy
Grados et al., 2000 [10]	1/40 (2.5%)	PVP, osteoporotic fracture	Not reported
Bernhard et al., 2003 [11]	1	PVP, osteoporotic fracture	Not reported
Pleser et al., 2004 [12]	1	PVP, osteoporotic fracture	Heparin + warfarin for 6 months
Seo et al., 2005 [13]	1	PVP, osteoporotic fracture	Operative embolectomy
Baumann et al., 2006 [14]	1	PVP, osteoporotic fracture	Warfarin for 3 months
Freitag et al., 2006 [15]	1	PVP, osteoporotic fracture	Warfarin for 6 months
MacTaggart et al., 2006 [16]	1	PVP, osteoporotic fracture	Not reported
Neuwirth et al., 2006 [17]	1	PVP, osteoporotic fracture	Not reported
Walz et al., 2006 [18]	1/57 (2%)	PVP, osteoporotic fracture	No anticoagulation
Quesada and Mutlu, 2006 [19]	1	PVP, osteoporotic fracture	Not reported
Abdul-Jalil et al., 2007 [20]	1	PVP, osteoporotic fracture	Low dose heparin
Serra et al., 2007 [21]	3/175 (2%)	PVP, osteoporotic fracture	Not reported
Schneider and Plit, 2007 [22]	1	PVP, osteoporotic fracture	Not reported
Yeo et al., 2009 [23]	18/119 (15%)	PVP, osteoporotic fracture	Not reported
Venmans et al., 2008 [24]	11/299 (3%)	PVP, osteoporotic fracture	Not reported
Venmans et al., 2010 [25]	14/54 (26%)	PVP, various	Not reported
Fornell-Pérez et al., 2010 [26]	1	PVP, osteoporotic fracture	Clinical observation
Nesnídal et al., 2010 [27]	1	PVP, osteoporotic fracture	Clinical observation
Dash and Brinster, 2011 [2]	1	PVP, osteoporotic fracture	Open heart surgery
Luetmer et al., 2011 [28]	22/244 (9%)	PVP, various	Clinical observation
Tourtier and Cottez, 2012 [29]	1	PVP, osteoporotic fracture	Clinical observation

Total	84 cases	PVP, osteoporotic fracture (100%)	47% not reported 19% clinical observation 9% warfarin alone 5% heparin alone 5% heparin + warfarin 5% open heart surgery 5% embolectomy 5% no anticoagulation

PVP: percutaneous vertebroplasty.
